# Impact of Ceramic Composition and Thickness on Light Transmission in CAD/CAM Lithium Disilicate Materials: An In Vitro Study

**DOI:** 10.1155/ijod/7488948

**Published:** 2025-08-31

**Authors:** Carlos A. Jurado, Salahaldeen Abuhammoud, Austin Green, Kelvin I. Afrashtehfar, Silvia Rojas-Rueda, Abdulaziz Alhotan, Franciele Floriani

**Affiliations:** ^1^Division of Operative Dentistry, Department of General Dentistry, The University of Tennessee Health Sciences Center College of Dentistry, Memphis 38103, Tennessee, USA; ^2^Ponce Health Sciences University School of Dental Medicine, Ponce 00716, Puerto Rico; ^3^Department of Prosthodontics, The University of Iowa College of Dentistry and Dental Clinics, Iowa City 52242, Iowa, USA; ^4^Department of Reconstructive Dentistry and Gerodontology, School of Dental Medicine, University of Bern, Bern 3010, Switzerland; ^5^Oral Implantology Research Institute (OIRI), Dubai, UAE; ^6^Consultant in Prosthodontics, Abu Dhabi, UAE; ^7^Division of Dental Biomaterials, The University of Alabama at Birmingham School of Dentistry, Birmingham 35233, Alabama, USA; ^8^Department of Dental Health, College of Applied Medical Sciences, King Saud University, P.O. Box 10219, Riyadh 12372, Saudi Arabia

**Keywords:** CAD/CAM, dental ceramic, fully-crystallized, light transmission, precrystallized

## Abstract

This study investigates light transmission through five types of computer-aided design/computer-aided manufacturing (CAD/CAM) lithium disilicate ceramics, varying in thickness (0.50, 1.00, and 1.50 mm). A total of 150 specimens (10 per group) were fabricated using both traditional and novel ceramic materials: E.max CAD (traditional), n!ce Straumann and LiSi Block GC (fully-crystallized), and Amber Mill and Cerec Tessera (precrystallized). After polishing, light transmission was measured using a curing radiometer and surface microstructures were examined with scanning electron microscopy (SEM). The data were analyzed using two-way analysis of variance (ANOVA) with Tukey's post hoc tests. Results revealed that light intensity decreased as the ceramic thickness increased, regardless of the material type. Amber Mill (0.50 mm) exhibited the highest light intensity at 537 mW/cm^2^, followed by E.max CAD (475 mW/cm^2^) and n!ce Straumann (470 mW/cm^2^). In contrast, LiSi Block GC (1.50 mm) showed no light transmission (0 mW/cm^2^), with Cerec Tessera (60 mW/cm^2^) and E.max CAD (175 mW/cm^2^) also exhibiting low transmission at 1.50 mm. SEM analysis identified structural differences among the materials. These findings suggest that both the composition and thickness of CAD/CAM lithium disilicate ceramics significantly impact light transmission. Results revealed that material composition and thickness significantly influenced light transmission values, underscoring the importance of selecting appropriate ceramic type and thickness to optimize polymerization during light-cured resin cementation in clinical practice.

## 1. Introduction

Over the past decade, the integration of computer-aided design (CAD) and computer-aided manufacturing (CAM) has revolutionized dentistry, leading to more efficient and precise clinical workflows [1–3]. The CAD/CAM process begins with capturing a digital impression using an intraoral scanner, followed by designing the restoration with specialized software. Finally, the restoration is fabricated through subtractive milling techniques [4]. Initially used for simpler restorations such as inlays and onlays, recent advancements in CAD/CAM systems have expanded their capabilities to include more complex dental cases [5–7]. As a result, modern dental training programs now integrate CAD/CAM technology from the outset, ensuring that practitioners are proficient in using these advanced tools in daily practice [8]. Additionally, patients increasingly prefer digital workflows over traditional methods, as evidenced by several studies [9, 10].

CAD/CAM systems have significantly impacted the selection of dental ceramics, offering a range of materials including feldspathic porcelain, leucite, lithium disilicate, zirconia-reinforced ceramics, and hybrid materials combining ceramics with polymers [11]. The choice of ceramic depends on factors like mechanical properties, optical characteristics, and fabrication time [12]. CAD/CAM ceramic blocks can be partially or fully-crystallized; partially crystallized blocks require further heating, while fully-crystallized blocks are milled without additional firing but tend to cause more wear on milling burs [13–15]. These ceramics are categorized into glass-based, glass-infiltrated, and nonglass-based types, with varying levels of transparency and strength [16].

Among glass-based ceramics, lithium disilicate has gained prominence due to its excellent mechanical and esthetic properties [17–19]. Initially introduced in the early 2000s as IPS E.max CAD, this material has been extensively studied, with clinical trials demonstrating high success rates for posterior crowns [20, 21]. For example, a 4-year study showed a 96.3% success rate, while a decade-long trial reported an 83.5% survival rate for lithium disilicate crowns in posterior regions [22]. In terms of optical properties, studies comparing lithium disilicate to zirconia ceramics have consistently found that lithium disilicate offers superior translucency and opalescence [23]. These optical qualities are crucial for ensuring effective light transmission during the cementation of restorations, which can affect the polymerization of resin cements [24]. Given the clinical relevance of ensuring adequate polymerization of composite resin cements beneath ceramic restorations, it is essential to understand how ceramic thickness influences light transmission [25]. Previous studies have reported varying minimum light intensity thresholds—from 100 to over 300 mW/cm^2^—depending on the ceramic material, thickness, and curing protocol [26]. Therefore, a more in-depth review of these parameters is critical for determining clinically acceptable thicknesses that do not compromise polymerization efficacy.

In response to evolving clinical needs, newer systems have been developed, such as Amber Mill and Cerec Tessera, which are precrystallized, and n!ce Straumann and LiSi Block GC, which are fully-crystallized ceramics [27]. These newer materials offer enhanced convenience, as some do not require additional crystallization after milling [28]. However, despite claims of improved optical properties in these new ceramics, independent studies comparing the light transmission capabilities of traditional and novel lithium disilicate ceramics remain scarce [29]. Understanding these differences is clinically relevant, particularly in scenarios where successful light polymerization of resin-based luting agents through ceramic restorations is critical to long-term restoration success and bond durability. This study aims to assess the light transmission values of traditional E.max CAD and four novel lithium disilicate ceramics (Amber Mill, Cerec Tessera, n!ce Straumann, and LiSi Block GC) at varying thicknesses (0.50, 1.00, and 1.50 mm). The null hypothesis of this study is that there is no statistically significant difference in light transmission among the five CAD/CAM lithium disilicate ceramics—E.max CAD, Amber Mill, Cerec Tessera, n!ce (Straumann), and LiSi Block (GC)—at each evaluated thickness (0.50, 1.00, and 1.50 mm).

## 2. Materials and Methods

The sample size (*n* = 10) per group was based on previously published studies on translucency and light transmission of ceramic materials. A post hoc power analysis was performed using G^*⁣*^*∗*^^Power (version X.X), confirming that the sample size provided sufficient statistical power (1 − *β* = 0.85) to detect medium effect sizes (*f* = 0.4) at a significance level of *α* = 0.05. All measurements were performed by a single trained examiner who was blinded to the material groups. A second calibrated examiner independently verified 20% of the measurements for reproducibility. Intraexaminer reliability was assessed using the intraclass correlation coefficient (ICC) and values above 0.90 were considered acceptable.

A total of 150 flat specimens (10 per group) were prepared from five different A2 shade CAD/CAM lithium disilicate materials: the traditional chairside e.max CAD (Ivoclar Vivadent, Schaan, Liechtenstein), two novel precrystallized materials—Amber Mill (HassBio, Gangwon-do, South Korea) and Cerec Tessera (Dentsply Sirona, Charlotte, NC, USA)—and two novel fully-crystallized materials—n!ce Straumann (Straumann Group, Basel, Switzerland) and LiSi GC Block (GC Corporation, Tokyo, Japan). Specimens were fabricated at thicknesses of 0.50, 1.00, and 1.50 mm using a precision cutting machine (Isomet, Buehler, Lake Bluff, Illinois, USA), resulting in 15 distinct groups: e.max CAD at 0.50 mm (Em05), Amber Mill at 0.50 mm (Am05), n!ce Straumann at 0.50 mm (N!05), LiSi GC Block at 0.50 mm (Li05), Cerec Tessera at 0.50 mm (Ce05); e.max CAD at 1.00 mm (Em10), Amber Mill at 1.00 mm (Am10), n!ce Straumann at 1.00 mm (N!10), LiSi GC Block at 1.00 mm (Li10), Cerec Tessera at 1.00 mm (Ce10); e.max CAD at 1.50 mm (Em15), Amber Mill at 1.50 mm (Am15), n!ce Straumann at 1.50 mm (N!15), LiSi GC Block at 1.50 mm (Li15), and Cerec Tessera at 1.50 mm (Ce15).

Crystallization of the specimens was carried out according to the manufacturer's recommended protocols, with e.max CAD fired at 840–850°C, Amber Mill at 825°C, and Cerec Tessera at 760°C. In contrast, the fully-crystallized materials, n!ce Straumann and LiSi GC Block, did not require any additional firing. The details of the ceramics and their components are listed in Table 1.

### 2.1. Light Transmission

The specimens underwent polishing using a lithium disilicate polishing kit (Brasseler LD, Brasseler USA, Savannah, GA, USA) in accordance with the manufacturer's guidelines. Subsequently, each sample's light transmission was assessed using an LED light curing unit (VALO Cordless, Ultradent, South Jordan, UT, USA), using a curing radiometer (Medical Instrument, Guilin, China) to record the average light intensity through the specimen in mW/cm^2^. The curing tip was maintained at a fixed distance of 1.0 mm from the specimen surface using a custom-built positioning jig. The curing light was applied perpendicularly (90° angle) to the surface to standardize angulation across all measurements. Light intensity was verified before each measurement session to ensure consistency.

### 2.2. Scanning Electron Microscopy (SEM) Images

The microstructure of each type of lithium disilicate ceramic was examined using scanning electron microscopy (SEM, TM 3000 M Hitachi High Technology, Tokyo, Japan). To facilitate electron conductivity during scanning, a coating of gold was applied to the specimen's surface using a sputter coater (Quick Coater Type SC-701, Sanyu Electro, Tokyo, Japan). This process allowed for the observation of the microstructure of the five different chairside CAD/CAM lithium disilicate ceramics.

### 2.3. Statistical Analysis

The normality of each variable was evaluated using the Shapiro–Wilk test. A parametric analysis was performed due to the exhibited normal distribution of the variables. The data underwent analysis using statistical software (SPSS ver.27, IBM, Armonk, NY, USA) with a two-way analysis of variance (ANOVA) set at a significance level of (*p*  < 0.05). Subsequently, a Tuckey HSD post hoc test was applied to discern light transmission differences among the five different materials available in three distinct thicknesses.

## 3. Results

Table 2 presents the mean light transmittance values (±standard deviation) for each lithium disilicate material at three thickness levels. At each thickness, ANOVA showed statistically significant differences among the materials (*p* < 0.001). Post hoc analysis (Tukey) confirmed that all materials differed significantly from one another (*p* < 0.05), as indicated by distinct letters in each row. At 0.50 mm, the highest transmittance was observed in e.max CAD (28.62 ± 1.47, Group Aa), followed by Amber Mill (27.78 ± 1.74, Ab), Cerec Tessera (26.74 ± 1.25, Ac), n!ce Straumann (25.06 ± 1.36, Ad), and LiSi Block (23.13 ± 1.32, Ae), with statistically significant differences among all materials (*p* < 0.05). As thickness increased to 1.00 mm, a similar descending pattern was maintained: e.max CAD (20.06 ± 1.06, Ba), Amber Mill (18.72 ± 1.19, Bb), Cerec Tessera (17.89 ± 0.87, Bc), n!ce Straumann (15.78 ± 1.08, Bd), and LiSi Block (13.63 ± 0.91, Be), again with significant differences (*p* < 0.05). At 1.50 mm, all materials showed reduced transmittance, with e.max CAD still highest (12.56 ± 0.93, Ca) and LiSi Block the lowest (6.35 ± 0.55, Ce). For specimens with a thickness of 0.50 mm, Amber Mill exhibited the highest values (537 mW/cm^2^), followed by e.max CAD (475 mW/cm^2^), n!ce Straumann (470 mW/cm^2^), and LiSi GC Block (405 mW/cm^2^). However, the lowest values were exhibited by Cerec Tessera (395 mW/cm^2^). In the case of ceramics with a 1.00 mm thickness, n!ce Straumann demonstrated the highest translucency (354 mW/cm^2^), followed by e.max CAD (320 mW/cm^2^), Amber Mill (255 mW/cm^2^), Cerec Tessera (220 mW/cm^2^), and the lowest translucency was noted with LiSi GC Block (200 mW/cm^2^). For ceramic with a thickness of 1.50 mm, the highest values were shown by n!ce Straumann (230 mW/cm^2^), followed by Amber Mill (205 mW/cm^2^), e.max CAD (175 mW/cm^2^), Cerec Tessera (60 mW/cm^2^), and LiSi GC Block, which showed no light transmission (0 mW/cm^2^).

**Figure 1:**
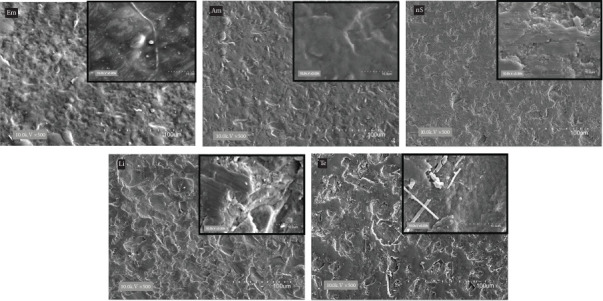
Scanning electron microscope (SEM) images of five CAD/CAM lithium disilicate ceramics: e.max CAD (Em), Amber Mill (Am), n!ce Straumann (nS), LiSi GC Block (Li), and Cerec Tessera (Te), captured at 500x magnification. The images highlight differences in surface morphology and crystalline structure among the materials.

Scanning electron microscope (SEM) analysis revealed distinct microstructural characteristics among the five tested lithium disilicate ceramics. E.max CAD and Amber Mill exhibited elongated lithium disilicate crystals with average lengths ranging from 0.8 to 1.5 µm, embedded in a glassy matrix, resulting in relatively smooth and homogeneous surfaces. Cerec Tessera showed a dense arrangement of needle-like crystals approximately 0.3–0.7 µm in length, dispersed within a zirconia-enriched glass matrix. The fully-crystallized materials, n!ce Straumann and LiSi GC Block, presented compact crystalline structures with randomly oriented crystals typically below 0.5 µm, even in rougher regions of the surface Figure 1. These morphological differences in crystal size and distribution may influence translucency behavior and light transmission properties.

## 4. Discussion

Chairside CAD/CAM ceramics have gained popularity among dental professionals due to their rapid fabrication capabilities and excellent esthetic and functional outcomes [30–33]. Among available options—such as feldspathic porcelain, leucite-reinforced ceramics, zirconia, and lithium disilicate—lithium disilicate remains one of the most widely used materials for anterior restorations due to its superior optical performance [27, 34–36]. Its translucency and esthetic potential have been validated in several clinical and laboratory studies [37–40].

This study assessed LED light transmission in five chairside CAD/CAM lithium disilicate ceramics: one traditional pre-crystallized ceramic (e.max CAD) and four novel systems—two pre-crystallized (Amber Mill and Cerec Tessera) and two fully-crystallized (n!ce Straumann and LiSi GC Block). Three clinically relevant thicknesses were evaluated: 0.50, 1.00, and 1.50 mm. Results confirmed that both ceramic type and thickness significantly influenced light transmission, with reduced transmission values observed as thickness increased. Therefore, the null hypothesis was rejected across all thickness levels.

Importantly, the SEM analysis provided insight into the microstructural basis of these optical differences. For example, Cerec Tessera demonstrated a distinct microstructure with dense, needle-like lithium disilicate crystals embedded in a zirconia-enriched matrix. This morphology likely contributes to increased light scattering, which aligns with its lower translucency values, especially at greater thicknesses. Conversely, e.max CAD and Amber Mill showed smoother microstructures with fewer irregularities, corresponding to more favorable light transmission. These findings reinforce how ceramic morphology directly influences light propagation, absorption, and scattering within the material.

In terms of clinical implications, understanding light transmission is critical for ensuring adequate polymerization of resin-based luting agents during cementation. Studies have demonstrated that irradiance below 100 mW/cm^2^ may compromise polymerization [41], and composites may require even higher values (≥300 mW/cm^2^) [42]. In our study, only LiSi GC Block (0 mW/cm^2^) and Cerec Tessera (60 mW/cm^2^) at 1.50 mm thickness fell below the 100 mW/cm^2^ threshold. At 1.00 mm, several materials also transmitted less than 300 mW/cm^2^. Thus, for restorations thicker than 1.00 mm, clinicians should be cautious when relying on light-cured-only resin cements and may consider dual-cure systems or limiting material thickness.

All specimens were standardized to A2 shade, one of the most commonly used in clinical practice. Shade significantly influences light transmission, as darker shades tend to scatter and absorb more light [42, 43, 44]. By using a single, clinically common shade, this study minimizes confounding from chromatic variation and ensures broader relevance.

While the results are promising, this study is not without limitations. First, only five CAD/CAM lithium disilicate ceramics were evaluated, and although they are representative, the rapidly evolving market includes additional systems that merit future evaluation. Second, flat disc specimens were used, which—while necessary for standardizing measurements—do not fully replicate the geometry of real dental restorations such as crowns or veneers. Light interaction with curved surfaces, marginal thicknesses, and surface textures can differ considerably in clinical scenarios. In summary, both the composition and thickness of CAD/CAM lithium disilicate ceramics play a significant role in their light transmission properties. These findings underscore the importance of material selection and restoration design in achieving adequate resin cement polymerization, particularly when using light-cured-only protocols.

Despite the controlled in vitro design, this study has several limitations. Firstly, only five chairside CAD/CAM lithium disilicate systems were evaluated, even though a broader array of products is available in the market. Secondly, flat, uniform disc specimens were used to standardize measurements; however, they do not replicate the complex geometry of clinical restorations such as crowns or veneers. In real-world applications, factors like anatomical contours, varying marginal thicknesses, and curved surfaces can influence how light propagates through the ceramic, potentially affecting polymerization outcomes. Thirdly, the study was conducted under idealized laboratory conditions that do not fully account for intraoral variables such as ambient light, temperature, humidity, and operator variability. Lastly, the light transmission values were not correlated with actual resin cement polymerization depth or hardness, which may limit the clinical extrapolation of the findings. Future studies using anatomically shaped crowns or veneers would enhance external validity. Additionally, variations in intraoral conditions, such as humidity and temperature, were not simulated in this in vitro design.

## 5. Conclusions

This study demonstrated that light transmission through CAD/CAM lithium disilicate ceramics decreases significantly with increased thickness, regardless of the material brand. However, within the same thickness, substantial differences in light transmission were observed among the materials, highlighting the influence of ceramic composition and microstructure. Amber Mill showed the highest transmission at 0.50 mm, whereas n!ce Straumann exhibited superior performance at both 1.00 and 1.50 mm thicknesses. These variations are primarily attributed to differences in crystal structure, filler content, and the proportion of the glassy matrix. Materials characterized by smaller crystal sizes, lower crystalline volume, and higher glass content—such as Amber Mill and n!ce Straumann—facilitated greater light transmission. In contrast, ceramics with higher crystalline filler concentrations or additional opacifying agents attenuated light more effectively. Therefore, both thickness and composition are critical factors influencing optical behavior and must be carefully considered in clinical decision-making. When light-cured resin cements are used, selecting a ceramic that allows sufficient light transmission is essential to ensure optimal polymerization and long-term clinical success.

## Figures and Tables

**Table 1:** Dental ceramics and their components used in this study.

Brand	Manufacturer/location	State	Main components	Mechanical properties
e.max CAD	Ivoclar Vivadent (Schaan, Liechtenstein)	Precrystallized	57%–80% SiO_2_, 11%–19% Li_2_O, 0%–13% K_2_O, 0%–11% P_2_O_5_, 0%–8% ZrO_2_, 0%–8% ZnO, other oxides	Flexural strength: 360–400 MPa; Elastic modulus: ~95 GPa;
Amber Mill	Hassbio (Gangwon-do, South Korea)	Precrystallized	SiO_2_ <78%, Li_2_O <12%, coloring oxides <12%	Flexural strength: ~400 MPa; Elastic modulus: ~90–100 GPa
n!ce	Straumann Group (Basel, Switzerland)	Fully-crystallized	64%–70% SiO_2_, 10.5%–12.5% Li_2_O, 0%–3% K_2_O, 3%–8% P_2_O_5_, 0%–0.5% ZrO_2_, 10.5%–11.5% Al_2_O_3_, 1%–2% CaO, 0%–9% pigments, 1%–3% Na_2_O	Flexural strength: ~400 MPa; Elastic modulus: ~90–95 GPa
LiSi GC Block	GC Corporation (Tokyo, Japan)	Fully-crystallized	55%–80% SiO_2_, 10%–30% Li_2_O, 5%–20% other oxides	Flexural strength: 370–400 MPa; Elastic modulus: ~93 GPa
Cerec Tessera	Dentsply Sirona (Charlotte, NC, USA)	Precrystallized	Li_2_Si_2_O_5_ (90%), Li_2_PO_4_ (5%), Li_0.5_Al_0.5_Si_2.5_O_6_ (virgilite; 5%)	Flexural strength: 420–500 MPa (dual cure); Elastic modulus: ~100 GPa

**Table 2:** Light transmission values (mW/cm^2^) of pre- and fully-crystallized chairside CAD/CAM lithium disilicate ceramics with 0.5, 1.00, and 1.50 mm thickness.

Thickness	e.max CAD	Amber Mill	n!ce Straumann	LiSi GC Block	Cerec Tessera
0.50 mm	475 (23.6)^Aa^	537 (48.9)^Ba^	470 (22.9)^Aa^	405 (24.6)^Ca^	395 (26.2)^Ca^
1.00 mm	320 (19.7)^Ab^	255 (43.8)^Bb^	345 (24.1)^Ab^	200 (23.6)^Cb^	220 (25.8)^Cb^
1.50 mm	175 (26.3)^Ac^	205 (28.4)^ABc^	230 (25.8)^Bc^	0 (0)^Cc^	60 (17.5)^Dc^

*Note:* Superscript letters indicate statistical difference.

## Data Availability

The data presented in this study are available upon request from the corresponding authors.
